# Youth and emotional vulnerability predict chatbot appeal

**DOI:** 10.3389/fpsyg.2026.1983308

**Published:** 2026-10-14

**Authors:** Dara Greenwood, Jingyi Shang

**Affiliations:** Vassar College, Poughkeepsie, NY, United States

**Keywords:** age, AI, attachment anxiety, chatbot, loneliness

## Abstract

The present survey study (Prolific sample, *N* = 361) aimed to clarify relationships among age and socioemotional well-being as they pertain to chatbot use and perception by sampling across two age cohorts: emerging adults (18–24 years) and middle-aged adults (48–54 years). We assessed loneliness and attachment anxiety in concert with AI chatbot use frequency, perceptions of chatbots as comforting, and as easier to interact with than humans. Results show that the younger cohort reported significantly more frequent chatbot use and increased perceived comfort from that use, compared to the older cohort. The younger cohort also reported significantly greater loneliness and attachment anxiety. Results of regression analyses show that attachment anxiety and loneliness each positively predicted perceptions of chatbot ease. The latter association was qualified by a marginal interaction with age: loneliness significantly predicted increased chatbot ease for the younger cohort only. Findings underscore the need to continue probing the appeal of chatbots for vulnerable populations and the potential impact those interactions may have on well-being.

## Introduction

As AI chatbot use continues apace—at the time of writing, OpenAI’s ChatGPT had reached *one billion* monthly users (Reuters, 2026)—it behooves psychological scientists to continue clarifying the demographic and psychological profiles of chatbot users, as well as the perceived gratifications of such use. Because chatbots can mimic many of the qualities that drive and enrich human interaction (e.g., empathy, reciprocity), it is perhaps not surprising that people have started to use them for socioemotional purposes in addition to practical ones (De Freitas, 2026). A recent survey found that although 42% of adults who say they use chatbots do so for information-seeking purposes, 10% report using them for emotional support and another 4% report using chatbots for companionship (Gottfried et al., 2026). However, these numbers obscure patterns of chatbot use that are relevant to both emotional well-being and age, which the present study is poised to probe.

Research has found that individuals who experience a deficit in their socioemotional lives may be more likely to utilize chatbots in socially and emotionally relevant ways. Specifically, motivation to use or appreciation for companionship-oriented chatbots has been predicted by attachment anxiety (worrying about the strength and stability of close relationships, e.g., Yang, 2026), loneliness (Latikka et al., 2026), and lower social network support (Herbener and Damholdt, 2025; Zhang K. et al., 2025; Zhang Y. et al., 2025). It stands to reason that if one’s socioemotional needs are not being met—due to relational insecurity, isolation, or lack of social support—one might gravitate toward a functional alternative, albeit a non-human one. Whether or not such use is beneficial for and/or harmful for mental health is a critical issue facing parents, clinicians, and AI companies alike and is beyond the scope of the present paper. However, a key piece of that puzzle is understanding the appeal and use of chatbots among potentially vulnerable populations.

Younger adults also constitute a type of vulnerable population; emerging adults (typically in the 18 to 25 age range) have been described as inhabiting an “age of instability” (Arnett and Tanner, 2006)—their lives are typically marked by transitions across key life domains from romance to employment. Accompanying this transitional phase is an increased likelihood of experiencing loneliness and attachment anxiety (Charles et al., 2023). Additionally, research shows that about a third of 18–29 year olds report using chatbots daily, a number which drops to 19% for those in the 50–64 age range, and to 7% for those 65 and older (Gottfried et al., 2026). Given young adults’ increased use of technology coupled with their increased emotional vulnerability, it is intuitive that a study of 18–28 year olds found that one third of them reported using AI for advice and about a quarter reported using AI for friendship (Lira et al., 2026). Another survey of 18–21 year olds found that 22% reported seeking AI’s help with mental health related issues (McBain et al., 2025).

The specific ways in which age and emotional well-being intersect to predict chatbot use remains unclear, however. Research focused on emotional health and chatbot use tends to sample from just one side of the aging spectrum (Herbener and Damholdt, 2025; Lira et al., 2026; McBain et al., 2025), or to treat age as a covariate (e.g., Zhang Y. et al., 2025; Zhang K. et al., 2025). The present study deliberately samples across two age cohorts—emerging adults (18–24) and middle-aged adults (48–54)—to clarify the relationships among age, emotional vulnerability, and chatbot use and perception. Importantly, we endeavored to examine age as a potential moderator between emotional well-being and chatbot use. We chose these two age cohorts because they represent distinct socioemotional stages as well as distinct AI adoption profiles. As noted above, middle-aged adults use AI less then emerging adults but more than older adults.

We first aim to replicate the finding that younger people report greater use of chatbots overall (H1a) and additionally predict that young adults, compared to older adults, will perceive chatbots as more comforting (H1b) and easy to interact with (H1c). We also expect, in line with prior work, that the younger cohort will report increased emotional vulnerability compared to the older cohort, as manifested in increased loneliness (H2a) and increased attachment anxiety (H2b). And, in line with work suggesting that companion-oriented chatbot use is more appealing for those with deficits in their socioemotional lives, we predict that loneliness and attachment anxiety will be associated with increase perceived comfort (H3a, H3b) and ease of chatbot use (H3c, H3d), irrespective of age.

Research on chatbot frequency *per se* and emotional well-being is thus far equivocal. For example, a study of Dutch adolescents finds no relationship between frequency of chatbot use and loneliness (Herbener and Damholdt, 2025), while other research finds intensity of use (frequency of use combined with emotional connections to chatbots) to be associated with *increased* well-being (controlling for age, Zhang Y. et al., 2025). Because of the mixed findings noted above regarding chatbot use frequency and emotional well-being, we ask: what are the relationships between loneliness, attachment anxiety and chatbot use frequency (RQ1, RQ2)?

Finally, given evidence that young people in particular may be more likely to use chatbots for social and emotional purposes, we predict that age will moderate the associations between attachment anxiety, loneliness and chatbot comfort (H4a, H4b) as well as chatbot ease (H4c, H4d). And, as a follow-up to the research questions about chatbot use frequency and well-being, we ask whether age will moderate between attachment anxiety and/or loneliness and chatbot frequency (RQ3, RQ4).

## Method

### Transparency and openness

All analyses were undertaken using standard SPSS syntax as well as the Hayes process macro (v. 5) in SPSS for running moderated regression analyses. Data and measures for this study will be made available upon request (the informed consent noted that the data would be restricted to research staff). This study was not pre-registered. An attempt was made to recruit 200 participants for each age cohort, prior to exclusions resulting in *n* = 361 participants (see Participants section for details as well as post-hoc power analysis). The measures utilized for this study were drawn from a larger survey that included other items broadly related to media and AI use that were not the focus of the current study.

### Participants

Participants were drawn from a U. S. Prolific sample for two age cohorts (18–24 years; 48–54 years) in December of 2025. The final sample (*N* = 361; *n* = 179 in the younger cohort and *n* = 182 in the older cohort) included those who said they ever used AI chatbots^1^ (52% male, 47% female, three nonbinary or gender non-conforming, one preferred not to answer). Post-hoc power analyses conducted on the final sample (Faul et al., 2009) reveal that, for example, for the moderated regression analyses based on sample sizes of at least *n* = 357, power was perfect (1.00) to detect a moderate effect size of *f*^2^ = 0.15.

Two-thirds (66%) of the sample identified as White/Caucasian, 17% as Black/African American, 12% as Asian/Asian American, 4% as bi/multiracial, 2% as Native American, and 2 participants as Native Hawaiian or other Pacific Islander (numbers exceed 100 due to rounding).

### Measures

#### Loneliness

We utilized the UCLA short-form loneliness scale (Hughes et al., 2004), comprised of three items assessing how often (*Hardly ever*, *Sometimes*, *Often*) participants experienced feeling lonely (e.g., “how often do you feel you lack companionship?” α = 0.88). Scores were obtained by summing across each of the three items.

#### Attachment anxiety

We used the attachment anxiety subscale of Collins (1996) Revised Adult Attachment Scale designed for general close relationships. For this 6-item scale (e.g., “I often worry that other people do not really love me,” α = 0.92), responses were made on a 5-pt Likert scale (*Not at all characteristic of me* to *Extremely characteristic of me*).

#### Chatbot frequency

Frequency of using chatbots was operationalized by a single item asking how often, if ever, participants used AI chatbots (e.g., ChatGPT, Claude, Gemini), ranging from *Never* to *Very often* (1–5). As noted earlier, only participants who reported ever using chatbots (2–5) were included.

#### Chatbot perceptions

Three items (drawn from Zhang K. et al., 2025) were employed to assess comfort derived from chatbot use (chatbot comfort, e.g., “chatbots are a great source of emotional support for me,” α = 0.91) and three items (drawn from Zhang Y. et al., 2025) asked about perceiving chatbots to be easier to interact with than humans (chatbot Ease, e.g., “I feel like I can be more open when communicating with chatbots,” α = 0.93). Both scales were assessed on a 1–7 scale (*Disagree strongly* to *Agree strongly*).

To ensure that chatbot comfort and chatbot ease were distinct constructs, we compared the results from two models in a confirmatory factor analysis (CFA). One model contained two latent factors (three items each as observed manifest variables), whereas the second model contained a single latent factor with all six items as manifest variables. The two-factor model demonstrated very good fit to the data, χ^2^ (8) = 17.68, *p* = 0.024, CFI = 0.99, TLI = 0.99, RMSEA = 0.06, SRMR = 0.02. In contrast, the one-factor model demonstrated substantially poorer fit, χ^2^ (9) = 436.62, *p* < 0.001, CFI = 0.76, TLI = 0.61, RMSEA = 0.36, SRMR = 0.11. Comparison of the nested models indicated that the two-factor model fit significantly better than the one-factor model, Δχ^2^(1) = 418.94, *p* < 0.001, ΔCFI = +0.23, ΔTLI = +0.38, ΔRMSEA = −0.30, ΔSRMR = −0.09.

### Procedure

The survey was deployed online via Qualtrics. Measures were completed in the order in which they are described above. Other measures of AI use and well-being were included in the larger survey but are not germane to the present study. A demographics section was included at the end of the survey to assess age, gender and ethnicity.

## Results

### Preliminary analyses

Preliminary analyses to probe gender differences across key study constructs revealed gender differences in chatbot frequency only: *t*(355) = 3.16, *p* = 0.002, *Cohen’s d* = 0.98. Specifically, males reported greater chatbot frequency than females, *M* = 3.75 (*SD* = 0.98) > *M =* 3.42 (*SD* = 0.99). Thus, we include gender as a binary covariate (excluding three nonbinary participants and one who preferred not to answer) in relevant analyses below.

### Age differences in chatbot use and emotional well-being

To assess whether younger participants would report increased chatbot frequency (H1a), chatbot comfort (H1b), and chatbot ease (H1c), we performed a MANCOVA (covarying gender). Results reveal an overall effect of age, *F*(3, 352) = 3.29, *p* = 0.021, 𝜂^2^ = 0.027. Inspection of univariate effects reveal support for H1a and H1b: the younger cohort scored significantly higher on both chatbot frequency, *F*(1, 354) = 8.90, *p* = 0.003, and perceptions of chatbot comfort, *F*(1, 354) = 4.68, *p* = 0.031. No differences in chatbot ease as a function of age emerged; H1c was not supported.

Results of a second MANOVA show an overall effect of age on emotional well-being, *F*(2, 358) = 25.14, *p* < 0.001, 𝜂^2^ = 0.123. Inspection of univariate effects indicate that younger participants scored significantly higher on loneliness, *F*(1, 359) = 16.09, *p* < 0.001 and attachment anxiety, *F*(1, 359) = 50.41, *p* < 0.001, in support of H2a and H2b. See Table 1.

**Table 1:** Mean comparisons of study variables across age cohort.

Variable (Scale Range)	Younger cohort (*n* = 179) *M* (*SE*)	Older cohort (*n* = 182) *M* (*SE*)	Cohen’s *d*
Chatbot frequency (1–5)	3.75 (0.07)	3.44 (0.07)**	0.98
Chatbot comfort (1–7)	3.50 (0.13)	3.11 (0.13)*	1.7
Chatbot ease (1–7)	4.01 (0.14)	3.75 (0.14)	1.9
Attachment anxiety (1–5)	3.01 (0.08)	2.25 (0.08)***	1.0
Loneliness (3–9)	5.68 (0.14)	4.88 (0.14)***	0.63

***p < 0.001; **p < 0.01; *p < 0.05; sample size varies slightly for the chatbot variables due to gender exclusions for the MANCOVA (*n* = 176 for younger cohort; *n* = 181 for the older cohort).

### Emotional well-being and chatbot use as a function of age

We next examined the predicted associations between loneliness, attachment anxiety and chatbot engagement (H3a-d) by running partial correlations (controlling for age cohort). No significant associations emerged with chatbot comfort. Chatbot ease was positively associated with loneliness, *r*(358) = 0.11, *p* = 0.036, and attachment anxiety, *r*(358) = 0.15, *p* = 0.005. Thus, H3c and H3d were supported. We additionally covaried gender when examining associations between emotional vulnerability and chatbot frequency (RQ1 & RQ2); however, no significant associations emerged.

To interrogate predicted or queried associations between emotional vulnerability and chatbot frequency and perceptions as a function of age, we conducted six moderated regression analyses (Hayes, 2013, version 5.0, model 1). We predicted each of the three chatbot variables from each of the two psychological constructs, using age as a moderator. For the regression predicting chatbot frequency, we once again entered gender as a covariate (see Tables 2, 3).

**Table 2:** Regression models predicting chatbot frequency, chatbot comfort, and chatbot ease from age and attachment anxiety.

Predictors	Chatbot frequency *b* (SE)	*t*	CI (LL, UL)	Chatbot comfort *b* (SE)	*t*	CI (LL, UL)	Chatbot ease *b* (SE)	t	CI (LL, UL)
Gender (Chatbot Frequency only)	−0.32** (0.10)	−3.06	−0.52, −0.11						
Age	−0.14 (0.29)	−0.48	−0.71, 0.43	0.07 (0.50)	0.15	−0.91, 1.06	0.26 (0.55)	0.47	−0.83, 1.35
Attachment Anxiety	−0.01 (0.07)	−0.17	−0.15, 0.13	0.17 (0.12)	1.34	−0.08, 0.41	0.33 (0.14)*	2.43	0.06, 0.60
Age x Attachment Anxiety	−0.08 (0.10)	−0.78	−0.28, 0.12	−0.15 (0.18)	−0.84	−0.50, 0.20	−0.12 (0.19)	−0.62	−0.50, 0.26
*R*-square	0.06***			0.02+			0.03*		

***p < 0.001; **p < 0.01; *p < 0.05; + *p <* .10.

**Table 3:** Regression models predicting chatbot frequency, chatbot comfort, and chatbot ease from age and loneliness.

Predictors	Chatbot frequency (Model 2) *b* (SE)	*t*	CI (LL, UL)	Chatbot comfort (Model 2) *b* (SE)	*t*	CI (LL, UL)	Chatbot ease (Model 2) *b* (SE)	*t*	CI (LL, UL)
Gender	−0.32 (.10)**	−3.10	−0.52, −0.12						
Age	−0.19 (0.31)	−0.61	−0.79, 0.42	0.04 (0.53)	0.08	−1.01, 1.09	0.80 (0.59)	1.35	−0.36, 1.95
Loneliness	0.03 (.12)	0.25	−0.20, 0.26	0.07 (0.20)	0.33	−0.33, 0.47	0.61 (0.22)**	2.75	0.17, 1.05
Age x Loneliness	−0.07 (0.16)	−0.42	−0.39, 0.25	−0.25 (0.28)	−0.89	−0.81, 0.31	−0.55 (0.31)+	−1.75	−1.17, .07
*R*-square	0.05***			0.02			0.03*		

***p < 0.001; **p < 0.01; *p < 0.05.

When chatbot frequency was the criterion variable, attachment anxiety was the predictor, the overall model was significant, *F*(3, 352) = 5.16, *p* < 0.001. Inspection of coefficients showed that a significant association emerged for gender (male) only, *b* = −0.32 (*SE* = 0.10), *p* = 0.002. When loneliness was the predictor, the same pattern emerged; the overall model was significant, *F*(3, 352) = 4.80, *p* < 0.001 and a significant association with chatbot frequency emerged for gender (male), *b* = −0.32 (*SE* = 0.10), *p* = 0.002.

When chatbot comfort was the criterion variable, the model with attachment anxiety as a predictor was marginally significant overall, F(3, 357) = 2.15, p = .094. However, none of the individual predictors reached significance. The model with loneliness as a predictor did not reach significance. Therefore, we did not find support for H4a or H4b.

When chatbot ease was the criterion variable and attachment anxiety was the predictor, the model was significant, *F*(3, 357) = 3.36, *p* = 0.019. Inspection of coefficients revealed that higher levels of attachment anxiety were significantly and positively associated with perceptions of chatbot ease, *b* = 0.33 (*SE* = 0.14), *p* = 0.015. However, no interaction with age emerged (H4c was not supported). When loneliness was entered to predict of chatbot ease, the model was significant, *F*(3, 357) = 3.13 *p* = 0.026. Inspection of coefficients shows that similar to attachment anxiety, loneliness was positively associated with chatbot ease, *b* = .61 (*SE* = .22), *p* = 0.006. This relationship was qualified, however, by a marginal interaction between loneliness and age, *b* = −.55 (*SE* = .31), *p* = 0.081, *F*(1, 357) = 3.07, Δ*R*^2^ = 0.01. Conditional effects analysis show that for the younger cohort, the relationship between loneliness and chatbot ease was significant (LLCI = 0.17, ULCI = 1.05), whereas for the older cohort, it was not (LLCI = −0.37, ULCI = 0.50). This provides tentative support for H4d. See Figure 1 for a graph of this interaction.^2^

**Figure 1:**
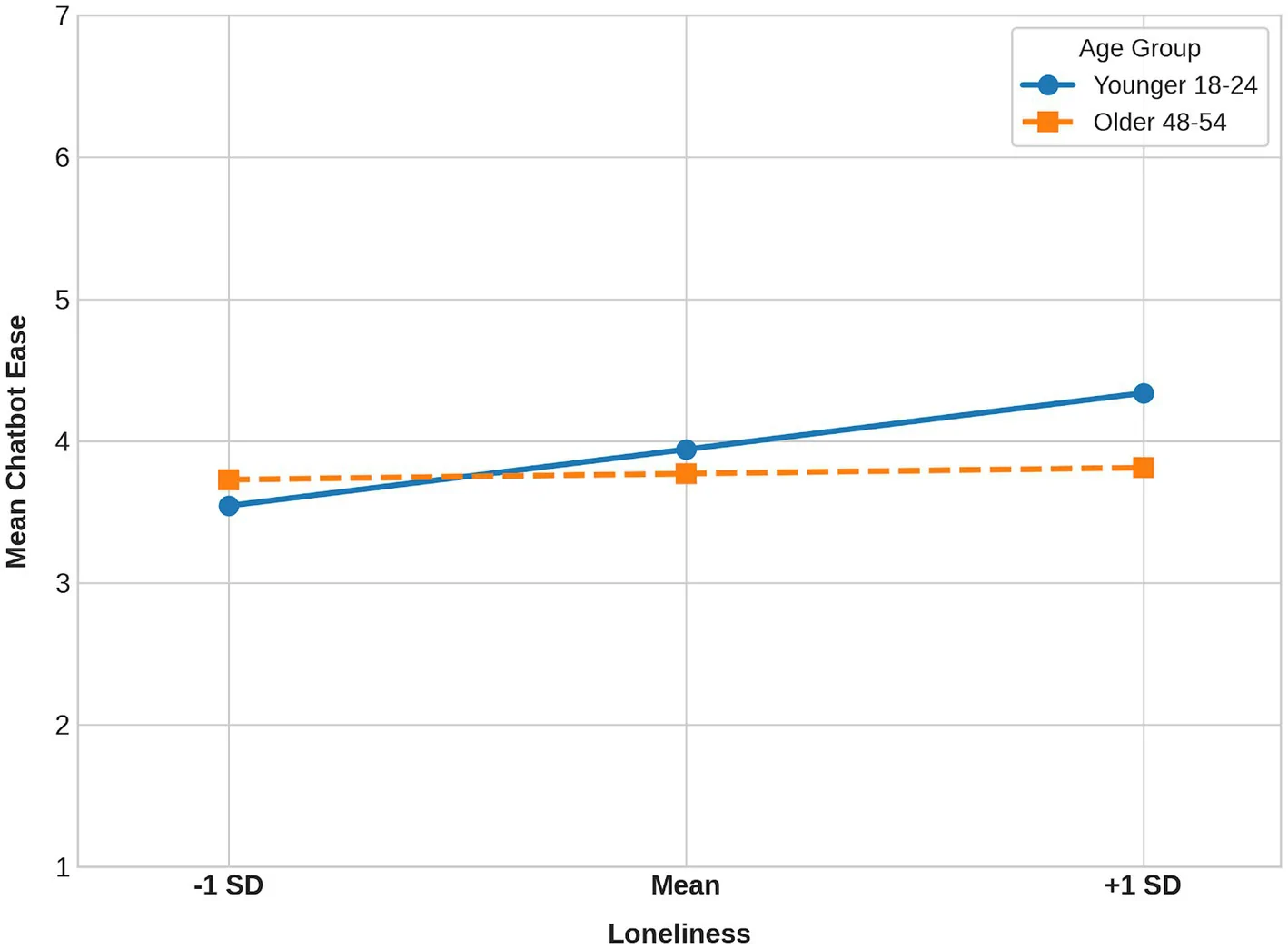
Interaction of age and loneliness on chatbot ease perceptions.

In sum, chatbot frequency was only significantly predicted by gender (male), chatbot comfort was not significantly predicted by age or emotional vulnerability, and chatbot ease was significantly predicted by both attachment anxiety and loneliness, the latter of which was marginally moderated by age in the predicted direction.

## Discussion

The present study replicates and extends our present understanding of how age and emotional well-being predict chatbot use and perceptions. In line with hypotheses, younger participants reported both increased emotional vulnerability as well as increased frequency of and comfort from chatbot use. Counter to predictions, however, there were no significant age differences for chatbot ease (e.g., “It is easier to disclose personal information to a chatbot”). Further, whereas loneliness and attachment anxiety were unrelated to either chatbot frequency or chatbot comfort perceptions, both were associated with perceived ease of interacting with chatbots in lieu of humans. In preliminary support of predictions, age marginally moderated the association between loneliness and chatbot ease; results showed that this association was significant for the younger cohort but not the older cohort.

Chatbot interactions may be perceived as particularly appealing for those whose increased relational anxieties may either inhibit them from turning to actual others for support or supersede others’ ability to provide needed reassurance. Although this may be adaptive in some circumstances and for some people, it may also dovetail into addictive tendencies. Some research shows that participants with high attachment anxiety are also more likely to report problematic use of conversational AI (Heng and Zhang, 2025). Further research is needed to understand when and for whom reliance on AI chatbots is beneficial.

For emerging adults, who utilize chatbots more frequently and find them more comforting than older adults, feelings of social isolation may increase the appeal of chatbots as low threat interaction partners. The absence of significant loneliness-linked effects among older adults may reflect their relatively increased socioemotional well-being and suggest that chatbot use in midlife may be more oriented around pragmatic than interpersonal use, as a recent survey found (Kakulla, 2026). This is aligned with older adults’ less frequent use of chatbots as well as their lower perceived comfort from that use.

Regression analyses showed that being male predicted frequency of chatbot use, in line with prior work (Latikka et al., 2026), but loneliness and attachment anxiety did not. This fits with previous work suggesting that frequency of chatbot use is unrelated to emotional well-being (Herbener and Damholdt, 2025). It was surprising that emotional vulnerability did not predict chatbot comfort (e.g., the perception that chatbots help one “feel not alone”). There may be a disconnect between the appeal of chatbots based on the relative ease of interaction they afford, and the *actual* emotional gratifications they provide. Although some research has found evidence that chatbot use reduces loneliness (De Freitas et al., 2026), other research on the use of chatbots for ersatz romance have noted the potential for “paradox of emotional connection to AI” (Li and Zhang, 2024, pp. 8–9)—namely, the tension between positive experiences of imagined intimacy and the realization that they are, in fact, not real. Still other work finds that feeling connected to a chatbot might come at the expense of feeling connected to one’s actual community (Gupta et al., 2025).

On the bright side of the AI coin, chatbots may function in therapeutically constructive ways, enabling individuals to mitigate their socioemotional deficits using an accessible and interactive computer-based partner. However, on the dark side of the coin is the possibility that chatbots are not, in fact, well-equipped for helping vulnerable individuals navigate more extreme mental health issues, as has been suggested by the recent disturbing cases of teen suicide that have occurred in the context of chatbot use (Chatterjee, 2025). Although AI developers have begun to partner with mental health professionals to optimize how chatbots engage with young people seeking emotional support (Entenberg, 2026), ongoing work is needed to assess when and for whom AI chatbot use functions as an adaptive vs. maladaptive tool.

Our study is limited by its correlational design and our focus on two age cohorts, as well as two particular indicators of well-being. More work is needed to examine how these trends manifest when age is considered as a continuous rather than discrete category. Further, ongoing work is needed to clarify the risks and benefits of conversational AI use among particular demographic and psychological profiles. Longitudinal research would be particularly useful for teasing out the motivations and impact of long-term engagement with AI chatbots. Ultimately, this research shows that both emotional vulnerability and youth are associated with an increased appeal of interacting with chatbots in lieu of humans.

## Data Availability

The raw data supporting the conclusions of this article will be made available by the authors, without undue reservation.

## Footnotes

1 ^2^Individuals excluded for never using AI chatbots were not significantly different in age, loneliness, or attachment anxiety than the group that ever-used chatbots. However, a chi-square test revealed that those who never used chatbots were more likely to be female (80% female) than male whereas a slight reverse pattern emerged for those who ever used chatbots (53% male), Χ^2^ (1, *N* = 392) = 13.84, *p* < 0.001.

2 The AI tool Gemini was used to create Figure 1 and was checked against data for validity.
